# Ralf Zimmermann and Luke Hanley (Eds.): Photoionization and photo-induced processes in mass spectrometry: fundamentals and applications

**DOI:** 10.1007/s00216-021-03426-0

**Published:** 2021-07-09

**Authors:** Jürgen H. Gross

**Affiliations:** Organisch-Chemisches Institut, Universität Heidelberg, Im Neuenheimer Feld 270, 69120 Heidelberg, Germany

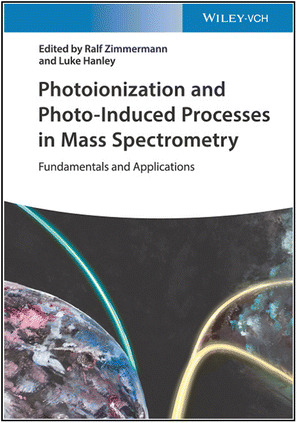



**Bibliography**

Photoionization and photo-induced processes in mass spectrometry: fundamentals and applications

Ralf Zimmermann and Luke Hanley (Eds.)

Wiley-VCH, Weinheim

ISBN: 978-3-527-33510-7

Hardcover, 421 pages

December 2020, €149.00

## Book’s topic

Photoionization (PI) can be effected by interaction of atoms or molecules with a single photon or with several photons, PI can be resonant or non-resonant, it can occur in high vacuum or at atmospheric pressure, and PI can be performed on gases and condensed matter alike. The photons of wavelengths from vacuum ultraviolet (VUV) to infrared (IR) are either delivered in short pulses by lasers or continuously by gas discharge lamps. This plethora of aspects from the fundamentals of PI processes to analytical applications is addressed in *Photoionization and photo-induced processes in mass spectrometry: fundamentals and applications*, edited by Ralf Zimmermann and Luke Hanley.

## Contents

This monograph comprises a total of eleven chapters of varying length. The first two chapters cover the fundamentals of vacuum photoionization and resonance-enhanced multiphoton ionization (REMPI), before chapters three and four deal with analytical applications of single-photon and resonance-enhanced multiphoton ionization. The next three chapters are dedicated to topics outside the analytical main stream: Chapter five deals with the application of synchrotron light sources, chapter six addresses elemental analysis by resonance ionization mass spectrometry (RIMS), and chapter seven introduces to femtosecond laser PI. The last four chapters cover more widespread analytical applications from atmospheric pressure photoionization to the fundamentals of laser desorption/ionization (LDI and MALDI), to laser desorption with postionization by a second laser, and finally to environmental analysis by single-particle mass spectrometry. Notably, the monograph contains 185 figures, the majority of them in color. The book also provides a table of contents and closes with a comprehensive subject index.

## Comparison with the existing literature

Photoionization has been the major topic of several books published since the 1970s but none of these appears comparable to the present monograph that comprises all ionization techniques in mass spectrometry involving photoionization. Moreover, nothing close to the present book seems to have been published during the last decades.

## Critical assessment

The chapters have been prepared by a collective of 16 authors, who have acted in teams of two, three, or in one case, four authors per chapter. Thus, some authors have contributed to two or more chapters; in particular, editor L. Hanley was involved in three and editor R. Zimmerman in five chapters. Obviously, this had an advantageous leveling effect on the quality of the chapters that comes very even across the entire book, and thus, pleasantly contrasts with those monographs where a multitude of authors tend to deliver quite inconsistently. Here, the editors managed to present a very consistent book in both level of scientific writing, i.e., concise explanations without being too short, and in the general appearance of figures that are evenly distributed across the chapters. The numerous figures (0.44 figures per page) also aid in the assimilation of the content. The references are uniformly cited in Harvard style, and thus, appear in alphabetical order of the first author at the end of each chapter. All journal references are given with article titles, thereby aiding in the judgement of their relevance.

There is not much to be criticized in this book. To be picky, one could mention that Figs. 7.8 and 7.9 are clearly below the otherwise high standard of the figures in both clarity of presentation and technical quality. And of course, this review would be incomplete without noting that Figs. 5.10, 6.11, 7.5 and 7.9 are erroneous in that they scale the abscissa using the incorrect *atomic mass unit* (amu, until 1961, based on oxygen) instead of the correct unified atomic mass (u, based on ^12^C, i.e., 1 amu ≠ 1 u). One also might suggest that chapters five to seven with their highly specialized topics could have better been shifted to the end of the monograph and that an author index would have been a worthy addition. Apart from this essentially minor critique, the book presents itself well very prepared and nicely rounded.

## Readership recommendation

This monograph can be recommended to researchers and practitioners in the field of mass spectrometry and to PhD students working with any type of photoionization. Due to its clear explanatory passages, it can also serve to introduce a broader readership to the many facets of photoionization.

## Summary

*Photoionization and photo-induced processes in mass spectrometry: fundamentals and applications* is a well-balanced book presenting an up-to-date monograph covering the entire range of topics related to this field. As promised in the subtitle, it combines explanatory sections on the underlying principles of photoionization processes and application-oriented chapters. Overall, it presents a highly recommended resource of the wide field of photoionization in mass spectrometry.

